# Tumor Regression Grade Predicts Survival in Locally Advanced Gastric Adenocarcinoma Patients with Lymph Node Metastasis

**DOI:** 10.1155/2020/3435673

**Published:** 2020-07-18

**Authors:** Yilin Tong, Yanmei Zhu, Yan Zhao, Zexing Shan, Jianjun Zhang, Dong Liu

**Affiliations:** ^1^Department of Gastric Surgery, Liaoning Cancer Hospital and Institute, Cancer Hospital of China Medical University, Shenyang, China; ^2^Department of Pathology, Liaoning Cancer Hospital and Institute, Cancer Hospital of China Medical University, Shenyang, China

## Abstract

**Background:**

Tumor regression grade (TRG) is widely used in gastrointestinal carcinoma to evaluate pathological responses to neoadjuvant chemotherapy (NCT), but whether it is an independent prognostic factor is still controversial. The aim of this study is to investigate the value of TRG in locally advanced gastric adenocarcinoma patients who underwent NCT and curative resection.

**Methods:**

Pathological regression was reevaluated according to the Mandard TRG. Survival curves were obtained by the Kaplan–Meier method, and differences in overall survival (OS) and disease-free survival (DFS) were compared using the log-rank test. Univariate and multivariate analyses for survival were based on the Cox proportional hazards method.

**Results:**

In total, 290 patients were identified in our electronic database. In univariable analysis, TRG was associated with OS (HR = 3.822, *P* ≤ 0.001) and DFS (HR = 3.374, *P* ≤ 0.001). However, in multivariable analysis, TRG was not an independent factor for OS (*P* = 0.231) or DFS (*P* = 0.191). In the stratified analysis, TRG retrieved prognostic significance in patients with the metastasis of lymph node (HR = 2.034, *P* = 0.035 for OS; HR = 2.220, *P* = 0.016 for DFS), while not in patients with negative lymph node (*P* = 0.296 for OS; *P* = 0.172 for DFS).

**Conclusions:**

TRG was not an independent predictor for survival, but the system regained its predicting significance in patients with lymph node metastasis.

## 1. Introduction

Neoadjuvant chemotherapy (NCT) has been successfully introduced in the management of gastrointestinal malignancies. It is recommended because of its potential benefits: downstaging of the primary tumor and lymph nodes [1], reducing tumor volume [2], facilitating complete surgical resection [3], treating systemic micrometastases [4], and improving survival [1].

Increasingly, the assessment of the effect of NCT becomes important. Tumor regression grade (TRG) is a system to evaluate the amount of residual tumor in patients who underwent preoperative therapy. Different from TNM stage, the TRG score focuses on the quantity rather than the location of the remain tumor, which could provide extra information on the response of tumor to the treatment and assist in predicting the prognosis [1].

There are many different grading systems without a global consensus [5–8]. Among numerous standards, the Mandard TRG, based on the relative amount between fibrosis and residual tumor, is the classic grading system and is widely applied in the gastrointestinal malignant carcinoma. The prognostic significance of this standard has been proved by many researches [1, 9, 10], but the boundaries to distinguish responders and nonresponders were not consistent. Some studies including the paper from the Mandard team suggested that patients with TRG 1-2 owned better survival than those with TRG 3-5 [5, 11, 12], while some other researches considered that TRG 1-3 vs. 4-5 had better predictive value [13–15]. Also, some studies supported utilizing three subgroups to describe prognosis was advantageous [10, 16].

In addition, in some cases, although TRG showed prognostic significance in univariable analysis, its prognostic worth was lost in multivariable analysis [3, 17–19]. Moreover, the metastasis of lymph node is always a great predictor for survival [3, 17–19]. And the relationship between the ypN stage and TRG was revealed by some studies [20–23]. So, it is reasonable to suppose that the metastasis of lymph node might partly mask the significance of TRG when analyzed simultaneously.

The purpose of this study is to validate the significance of the Mandard TRG system in locally advanced gastric adenocarcinoma patients, who undergone neoadjuvant chemotherapy and curative surgery based on our database, and explore if the significance of TRG would change in patients with different status of lymph node.

## 2. Materials and Methods

### 2.1. Patients

Patients with locally advanced gastric adenocarcinoma (including esophagogastric junction carcinoma) who underwent neoadjuvant chemotherapy in our institute between July 2010 and June 2016 were identified from the electronic database of our hospital. The eligibility criteria of our study included the following: (1) histopathological evidence of gastric adenocarcinoma; (2) locally advanced gastric cancer (8^th^ AJCC clinical stage: cT2N1M0~T4N3M0, II~III); (3) underwent neoadjuvant chemotherapy with or without postoperative treatment; (4) curative gastrectomy surgery was performed; and (5) age ranged from 20 to 80 years old. Exclusion criteria were as follows: (1) underwent preoperative radiotherapy; (2) suffering from other malignant tumor or gastric remnant cancer; and (3) incomplete information on staging or therapy.

### 2.2. Pathological Response Assessment

All the slides or blocks of surgical specimens were retrieved from the biospecimen library of our hospital and were reevaluated by two experienced gastrointestinal pathologists (YM.Z and D.L), respectively. The TNM stage was evaluated according to the 8^th^edition of the AJCC cancer staging. Histological regression grade of the primary tumor was assessed in accordance with the Mandard criteria: TRG 1 (fibrosis with no evidence of residual tumor, i.e., complete regression), TRG 2 (fibrosis with single cells or rare groups of residual tumor cells), TRG 3 (fibrosis and residual tumor with a dominance of fibrosis), TRG 4 (fibrosis and residual tumor with a dominance of tumor), and TRG 5 (extensive tumor without evidence of regression). When disagreement between pathologists appeared, a consensus would be reached by joint re-review and discussion through a multihead microscope. Other extracted histopathologic characteristics were reconfirmed during the assessment process.

### 2.3. Statistical Methods

Survival curves for overall survival (OS) and disease-free survival (DFS) were obtained using the Kaplan–Meier method, and the log-rank test was used to compare survival differences. The Cox regression analysis was used to assess the prognostic risk of clinicopathological characteristics on OS and DFS, and the factors with *P* value ≤ 0.05 or with great importance in clinical diagnosis were taken into multivariable analysis. OS was calculated from the time of initial treatment to death from any cause or last date of follow-up, while DFS was the time from the surgery to the date of recurrence or last follow-up day. *P* < 0.05 was considered statistically significant for all tests. Data was proceeded by the SPSS 25.0 software.

## 3. Results

### 3.1. Patient and Clinical Characteristics

From 3196 patients, 290 matched the criteria of our study, and their baseline characteristics are listed in Table 1. Most patients were male (74.1%). The median age was 59 years (range from 25 to 77). More than half number of tumors located in the lower third part (59.3%). A majority of patients underwent preoperative therapy of SOX (73.8%), and only a few refused to take adjuvant treatment (10.7%). The median time interval between completion of neoadjuvant treatment and surgery was 31 days, with an interquartile range of 28 to 36 days.

### 3.2. Pathological Features

The median number of reviewed slides indicating surgical specimen was 4 with an interquartile range from 3 to 5. After reevaluation, 9 patients had no residual tumor (ypT0). In 57 patients (19.6%), residual tumors did not extend beyond the muscular layer (ypT1-2). In 224 patients (77.3%), residual tumors reached or exceeded the subserosal layer (ypT3-4). 143 patients were intestinal classification (49.3%), while only 70 patients were well differentiated (25.2%). Vascular or lymphatic invasion (VOLI) was found in 72 patients (24.8%), while nervous invasion (NI) in 68 patients (23.4%).

**Figure 1:**
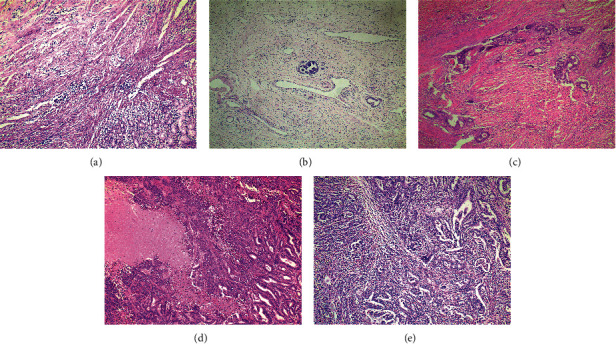
Examples of Mandard TRG. (a) Complete tumor regression, TRG 1. (b) Rare residual tumor, TRG 2. (c) More residual tumor but less than fibrosis, TRG 3. (d) Residual tumor with signs of regression, TRG 4. (e) Residual tumor without regression, TRG 5.

As for tumor regression grade, the example of every grade of Mandard TRG is shown in Figure 1. The number of patients in every group was 9, 84, 90, 85, and 22 for TRG 1-5, respectively.

The average number of lymph node removed was 28, with an interquartile range from 19 to 33. 100 patients had no lymph node metastasis (ypN-). 190 patients had at least one lymph node metastasis (ypN+), with the average number of positive nodes being 7.

### 3.3. Survival Analysis of TRG

The median follow-up for all patients was 41 months, with an interquartile range of 21 to 55 months. No significant difference in OS (*P* = 0.342) and DFS (*P* = 0.233) was observed between patients with TRG 1 and 2. For patients with TRG 3, 4, and 5, significant difference was found in DFS (*P* = 0.019), while not in OS (*P* = 0.170) (data not shown). Therefore, we divided the patients into two groups, TRG 1-2, defined as responders, and TRG 3-5, defined as nonresponders.

For 93 patients with TRG 1-2, the median OS and DFS were 52 and 50 months, respectively. For 197 patients in TRG 3-5 team, the median OS and DFS were 35 and 24 months, respectively. In univariable analysis, patients with TRG 1-2 owned better OS (HR = 3.822, *P* ≤ 0.001) and DFS (HR = 3.374, *P* ≤ 0.001) (Table 2). However, in multivariable analysis, TRG was not an independent prognostic factor for OS (HR = 1.429, *P* = 0.231) and DFS (HR = 1.430, *P* = 0.191) (Table 3).

In addition, the lymph node metastasis stage owned the highest hazard ratio among the independent prognostic factors for OS (all *P* ≤ 0.005) and DFS (all *P* ≤ 0.011) (Table 3).

### 3.4. Stratified Analysis by Lymph Node Metastasis

Because of the metastasis of lymph node possessing great impact on survival, which might influence the effect of TRG on prognosis, the stratified analysis by lymph node metastasis was performed.

**Figure 2:**
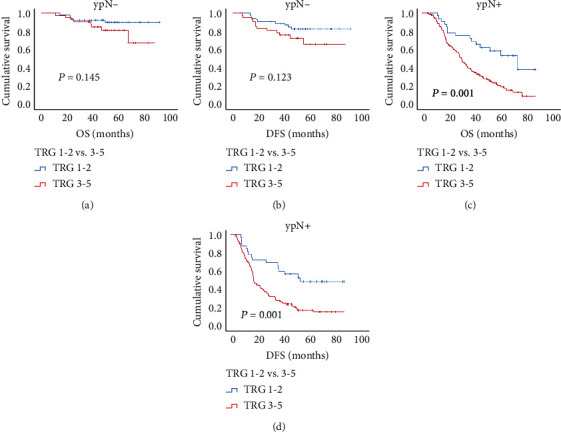
The Kaplan–Meier curves of grouped TRG stratified by ypN status. TRG lost predicting significance for OS (a) and DFS (b) in ypN- patients, while retrieved predicting significance for OS (c) and DFS (d) in ypN+ patients.

In the analysis of ypN- patients, survival curves showed TRG was not related to OS (*P* = 0.145) and DFS (*P* = 0.123) (Figure 2), and in multivariable analysis, TRG was still not an independent prognostic factor for OS (*P* = 0.296) and DFS (*P* = 0.172) (Table S1). However, in the ypN+ group, TRG showed markable prognostic significance in survival curves for OS (*P* = 0.001) and DFS (*P* = 0.001) (Figure 2). And, in multivariable analysis, TRG was an independent predictor for both OS (HR = 2.034, *P* = 0.035) and DFS (HR = 2.220, *P* = 0.016) (Table 4).

## 4. Discussion

As neoadjuvant therapy has been recommended to apply in gastrointestinal malignancy by various treatment guidelines in the world, the assessment of the effectiveness of preoperative therapy is increasingly important. Although the posttreatment TNM stage (ypTNM) has been widely accepted, this system is not always associated with prognosis in multivariable analysis, such as ypT stage [11, 20]. This might because the ypT stage is based merely on the location of residual tumor, which could not describe the remaining tumor completely. As a supplement, the tumor regression grading system, based on the quantity of residual tumor, is getting growing attention worldwide.

These systems could be classified into two mainly categories on the basis of comparison methods: compare the relative amount between fibrosis and residual tumor, such as the Mandard [5] and Ryan TRG [6], or appraise the proportion of the residual tumor in the previous tumor area, such as the Becker [7] and Rodel TRG [8]. Each category has been proved to associate with survival [1, 9, 24].

However, some disadvantages do exist in these systems. Firstly, there are numerous methods without universal agreement. According to a recently published survey from six continents, the standards applied were various in different countries [25]. This disparity might hinder the comparison among various researches.

Secondly, although this system is often an indicator to the prognosis in the univariable analysis, sometimes TRG lost its statistical significance in multivariable analysis [3, 17, 18]. The reason to this variation is still unclear. The contributors might include the connection or collinearity between TRG and other pathological factors [14, 21, 22], or the existence of other markable factors that cover up or divide the significance of TRG [3, 18, 19].

Thirdly, different researches using the same grading system supported different boundaries to keep the maximum predicting ability. This conflict is more obvious in the Mandard TRG, partly because of its higher number of tiers [16].

In our study, we confirmed the Mandard TRG was associated with OS and DFS in univariable analysis, especially merged into TRG 1-2 vs. 3-5. This boundary was in line with some studies [3, 11, 12] including the original one [5], but different with other studies, which supported TRG 1-3 vs. 4-5 [13–15]. Further studies are needed to explore if there is difference in survival between patients with TRG 2 and 3.

In multivariable analysis, we found TRG lost its prognostic significance, while the metastasis of lymph node owned the greatest hazard ratio. Therefore, the stratification analysis was performed to detect the role of TRG in patients with different lymph node status. The result showed the tumor regression grading system retrieved its prognostic significance in ypN+ patients while not in the ypN- group. On this point, a Japanese team found TRG was related to OS when a subset analysis of lymph node stage was conducted; however, they suggested this in patients with less than 7 positive nodes [19]. The discrepancy might partly result from their study was based on another regression system.

In addition to stratification, the combination of TRG and lymph node stage was also used to predict the survival [2, 3, 18, 26]. Based on the MAGIC trial, Smyth et al. [3] found that patients in TRG 3-5 and node positive group owned worse overall survival than others, while patients with TRG 1-2 and node negative were the best, which is consistent with our result. Another paper from the UK showed similar outcome, while they suggested the boundary should be TRG 1-3 vs. 4-5 [18]. Holscher et al. [26] and Martin-Romano et al. [2] also considered the responders with node negative owned better prognosis when using the TRG of proportion.

Except for the status of lymph node, other pathological factors including T stage [8, 22, 23], the Lauren classification [22, 27], the grade of differentiation [11, 22], and tumor type [28, 29] were also considered to correlate with TRG, which might affect the significance of this system. More studies are needed to investigate if the TRG system would show different significance on prognosis in patients with various clinicopathological characteristics.

There are some limitations in the present study. The study is retrospective and conducted at a single institution, which means a potential selection bias might exist. The sample size is small, which leads to a limited number of patients and excessive hazard ratios in the stratified analysis. The follow-up time is not long enough, which might hide the significance of TRG in lymph negative patients. But our study forced on a specific group of patients and confirmed the value of TRG in patients with lymph node metastasis. This suggested the system could contribute to the assessment of therapeutic effect and hinted that the significance of this system might be different according to clinicopathological characteristic of patients, which could partly account for the reason that TRG was not an independent predictive factor.

## 5. Conclusions

TRG was not an independent factor for survival, but the system regained its prognostic significance in the patients with lymph node metastasis. Therefore, the combined application of TNM and TRG system could make a contribution to the evaluation of the efficacy of neoadjuvant therapy.

## Figures and Tables

**Table 1:** Clinicopathological characteristics.

Characteristics	No. of patients	Percent
Gender		
Male	215	74.1
Female	75	25.9
Age		
<65	221	76.2
≥65	69	23.8
Tumor location		
L	172	59.3
M	54	18.6
U	32	11.0
GEJ	8	2.8
Diffuse	24	8.3
Tumor size (cm)		
<5	115	39.7
≥5	175	60.3
ypT		
0	9	3.1
1-2	57	19.6
3-4	224	77.3
ypN		
0	100	34.5
1	49	16.9
2	79	27.2
3	62	21.4
ypTNM		
I	52	17.9
II	71	24.5
III	167	57.6
Histological type		
Adenocarcinoma	186	64.1
Mucinous or signet ring cell carcinoma	104	35.9
Lauren classification		
Intestinal	143	49.3
Diffuse or mixed	147	50.7
Grade of differentiation		
Well	70	25.2
Moderate or poor	220	74.8
Vascular or lymphatic invasion		
No	218	75.2
Yes	72	24.8
Nervous invasion		
No	222	76.6
Yes	68	23.4
Mandard TRG		
1	9	3.1
2	84	29.0
3	90	31.0
4	85	29.3
5	22	7.6
Neoadjuvant therapy		
SOX	214	73.8
XELOX	21	7.2
FOLFOX	55	19.0
Adjuvant treatment		
No	31	10.7
Yes	259	89.3

**Table 2:** Univariate analysis of clinicopathological factors.

Prognostic factors	OS		DFS	
	Hazard ratio (95% CI)	*P* value	Hazard ratio (95% CI)	*P* value
Gender	1.298 (0.896, 1.882)	0.168	1.218 (0.854, 1.737)	0.276
Age	1.572 (1.084, 2.260)	0.017	1.445 (1.013, 2.062)	0.042
Tumor location		0.001		0.001
L	1		1	
M	0.727 (0.443, 1.191)	0.206	0.683 (0.424, 1.102)	0.118
U	1.098 (0.620, 1.945)	0.748	0.921 (0.523, 1.621)	0.776
GEJ	2.262 (1.041, 4.915)	0.039	2.251 (1.089, 4.653)	0.028
Diffuse	2.537 (1.511, 4.259)	0.001	2.636 (1.612, 4.309)	0.001
Tumor size (cm)	2.647 (1.784, 3.928)	0.001	2.198 (1.535, 3.148)	0.001
ypT		0.001		0.001
0	1		1	
1-2	1.968 (0.252, 15.379)	0.519	2.463 (0.322, 18.829)	0.385
3-4	8.166 (1.140, 58.501)	0.001	8.953 (1.251, 64.084)	0.029
ypN		0.001		0.001
0	1		1	
1	5.398 (2.860, 10.187)	0.001	3.338 (1.868, 5.966)	0.001
2	5.284 (2.873, 9.718)	0.001	4.291 (2.570, 7.165)	0.001
3	13.507 (7.383, 24.711)	0.001	9.483 (5.662, 15.884)	0.001
ypTNM				
I-II vs. III	9.214 (1.161, 73.009)	0.036	4.480 (3.005, 6.678)	0.001
Histological type	1.576 (1.117, 2.222)	0.010	1.471 (1.060, 2.041)	0.021
Lauren classification	2.223 (1.557, 3.174)	0.001	2.136 (1.528, 2.987)	0.001
Grade of differentiation	3.521 (2.023, 6.129)	0.001	3.315 (1.999, 5.498)	0.001
Vascular or lymphatic invasion	2.242 (1.568, 3.204)	0.001	2.133 (1.513, 3.005)	0.001
Nervous invasion	1.652 (1.142, 2.390)	0.008	1.610 (1.131, 2.291)	0.008
Mandard TRG				
1-2 vs. 3-5	3.822 (2.371, 6.162)	0.001	3.374 (2.190, 5.200)	0.001
Neoadjuvant therapy		0.344		0.627
FOLFOX	1		1	
SOX	0.895 (0.594, 1.350)	0.597	0.855 (0.576, 1.269)	0.436
XELOX	0.520 (0.216, 1.250)	0.144	0.728 (0.347, 1.526)	0.400
Adjuvant treatment	1.659 (0.996, 2.763)	0.052	1.790 (1.117, 2.871)	0.016

**Table 3:** Multivariate analysis of prognostic factors.

Prognostic factors	OS		DFS	
	Hazard ratio (95% CI)	*P* value	Hazard ratio (95% CI)	*P* value
Age	1.713 (1.154, 2.543)	0.008	1.474 (1.014, 2.145)	0.042
Tumor location		0.053		0.022
L	1		1	
M	0.669 (0.392, 1.143)	0.141	0.617 (0.369, 1.032)	0.066
U	1.526 (0.823, 2.832)	0.180	1.148 (0.630, 2.093)	0.652
GEJ	1.100 (0.491, 2.465)	0.818	1.192 (0.564, 2.519)	0.645
Diffuse	1.759 (0.993, 3.118)	0.053	1.919 (1.117, 3.297)	0.018
Tumor size (cm)	1.772 (1.102, 2.849)	0.018	1.556 (0.996, 2.430)	0.052
ypT		0.644		0.438
0	1		1	
1-2	1.204 (0.138, 10.500)	0.867	1.384 (0.167, 11.434)	0.763
3-4	1.954 (0.212, 17.971)	0.554	2.340 (0.281, 19.514)	0.432
ypN		0.005		0.011
0	1		1	
1	5.104 (1.733, 15.027)	0.003	3.317 (1.345, 8.184)	0.009
2	4.882 (1.618, 14.730)	0.005	3.600 (1.426, 9.088)	0.007
3	7.641 (2.404, 24.283)	0.001	5.200 (1.949, 13.869)	0.001
ypTNM	0.842 (0.266, 2.663)	0.769	0.747 (0.283, 1.974)	0.557
Histological type	0.934 (0.631, 1.382)	0.731	0.879 (0.605, 1.276)	0.498
Lauren classification	1.433 (0.942, 2.181)	0.093	1.334 (0.900, 1.979)	0.152
Grade of differentiation	1.620 (0.849, 3.093)	0.143	1.666 (0.915, 3.032)	0.095
Vascular or lymphatic invasion	1.739 (1.166, 2.592)	0.007	1.465 (1.002, 2.144)	0.049
Nervous invasion	0.966 (0.645, 1.447)	0.866	0.981 (0.668, 1.441)	0.924
Mandard TRG	1.429 (0.797, 2.561)	0.231	1.430 (0.836, 2.443)	0.191
Adjuvant treatment	2.556 (1.486, 4.396)	0.001	2.556 (1.531, 4.270)	0.001

**Table 4:** Multivariate analysis of patients with lymph node metastasis.

Prognostic factors	OS		DFS	
	Hazard ratio (95% CI)	*P* value	Hazard ratio (95% CI)	*P* value
Age	1.815 (1.197, 2.752)	0.005	1.396 (0.932, 2.091)	0.105
Tumor location		0.128		0.053
L	1		1	
M	0.626 (0.358, 1.094)	0.100	0.597 (0.342, 1.040)	0.068
U	1.196 (0.593, 2.413)	0.618	1.057 (0.532, 2.100)	0.873
GEJ	1.139 (0.511, 2.539)	0.750	1.260 (0.599, 2.651)	0.542
Diffuse	1.676 (0.932, 3.014)	0.085	1.809 (1.029, 3.012)	0.040
Tumor size (cm)	2.093 (1.276, 3.431)	0.003	1.864 (1.153, 3.012)	0.011
ypT		0.693		0.458
0	1		1	
1-2	0.907 (0.098, 8.431)	0.932	1.009 (0.112, 9.109)	0.994
3-4	0.366 (0.019, 7.221)	0.509	0.280 (0.015, 5.128)	0.391
ypTNM	2.432 (0.305, 19.371)	0.401	3.079 (0.424, 22.371)	0.266
Histological type	0.913 (0.603, 1.380)	0.665	0.808 (0.539, 1.213)	0.304
Lauren classification	1.517 (0.964, 2.389)	0.072	1.431 (0.919, 2.227)	0.113
Grade of differentiation	1.360 (0.673, 2.749)	0.392	1.228 (0.630, 2.393)	0.546
Vascular or lymphatic invasion	1.903 (1.265, 2.862)	0.002	1.635 (1.101, 2.427)	0.015
Nervous invasion	1.003 (0.662, 1.520)	0.988	0.993 (0.662, 1.490)	0.973
Mandard TRG	2.034 (1.052, 3.934)	0.035	2.220 (1.162, 4.241)	0.016
Adjuvant treatment	2.464 (1.393, 4.358)	0.002	2.339 (1.337, 4.092)	0.003

## Data Availability

The datasets analyzed during the current study are not publicly available due to the presence of identifiable patient information but are available from the corresponding author on reasonable request.

## Abbreviations

NCT: Neoadjuvant chemotherapy
GC: Gastric cancer
TRG: Tumor regression grade
VOLI: Vascular or lymphatic invasion
NI: Nervous invasion
OS: Overall survival
DFS: Disease-free survival
TNM: Tumor-node-metastasis
yp-: Postneoadjuvant therapy.

## Supplementary Materials

Supplementary Materials Table S1: multivariable analysis for patients with no lymph node metastasis.
