# Supplementary material for: Tumor Regression Grade Predicts Survival in Locally Advanced Gastric Adenocarcinoma Patients with Lymph Node Metastasis
Source: Gastroenterol Res Pract. 2020 Jul 18;2020:3435673. doi: 10.1155/2020/3435673 (PMC7383330; doi:10.1155/2020/3435673)
Supplement: Supplementary Materials — Table S1: multivariable analysis for patients with no lymph node metastasis. [file 3435673.f1.docx]

| Supplementary Table | Multivariable analysis for patients with no lymph node metastasis | | | |
| --- | --- | --- | --- | --- |
|  | OS |  | DFS |  |
| Prognostic factors | Hazard ratio (95% CI) | P value | Hazard ratio (95% CI) | P value |
| Age | 1.734 (0.385, 7.819) | 0.474 | 4.662 (1.463, 14.855) | **0.009** |
| Tumor location | | 0.449 |  | **0.038** |
| L | 1 |  | 1 |  |
| M | 0.409 (0.069, 2.435) | 0.326 | 0.349 (0.079, 1.536) | 0.164 |
| U | 0.997 (0.164, 6.055) | 0.998 | 0.434 (0.085, 2.219) | 0.316 |
| GEJ | - |  | - |  |
| Diffuse | 4.266 (0.338, 53.838) | 0.262 | 6.738 (1.079, 42.081) | **0.041** |
| Tumor size (cm) | 0.476 (0.091, 2.489) | 0.379 | 0.879 (0.249, 3.104) | 0.841 |
| ypT |  | **0.032** |  | **0.008** |
| 0 | 1 |  | 1 |  |
| 1-2 | * | 0.954 | * | 0.949 |
| 3-4 | * | 0.934 | * | 0.934 |
| ypTNM | 10.700 (0.663, 172.660) | 0.095 | 4.865 (0.427, 55.374) | 0.202 |
| Histological type | 1.695 (0.319, 9.008) | 0.536 | 1.497 (0.421, 5.324) | 0.533 |
| Lauren classification | 2.281 (0.470, 11.059) | 0.306 | 1.317 (0.431, 4.020) | 0.629 |
| Grade of differentiation | 1.498 (0.206, 10.887) | 0.690 | 3.777 (0.720, 19.811) | 0.116 |
| Vascular or lymphatic invasion | 1.535 (0.277, 8.518) | 0.624 | 1.262 (0.316, 5.046) | 0.742 |
| Nervous invasion | 0.167 (0.013, 2.078) | 0.164 | 0.637 (0.135, 3.009) | 0.569 |
| Mandard TRG | 0.416 (0.081, 2.151) | 0.296 | 0.404 (0.110, 1.485) | 0.172 |
| Adjuvant treatment | 0.493 (0.041, 5.869) | 0.576 | 1.967 (0.424, 9.114) | 0.387 |
| Note: *too large to record | |  |  |  |
